# Chondroitin sulfate efficacy versus celecoxib on knee osteoarthritis structural changes using magnetic resonance imaging: a 2-year multicentre exploratory study

**DOI:** 10.1186/s13075-016-1149-0

**Published:** 2016-11-03

**Authors:** Jean-Pierre Pelletier, Jean-Pierre Raynauld, André D. Beaulieu, Louis Bessette, Frédéric Morin, Artur J. de Brum-Fernandes, Philippe Delorme, Marc Dorais, Patrice Paiement, François Abram, Johanne Martel-Pelletier

**Affiliations:** 1 Osteoarthritis Research Unit, University of Montreal Hospital Research Centre (CRCHUM), 900 Saint-Denis, Suite R11.412, Montreal, Quebec H2X 0A9 Canada; 2 Institut de rhumatologie de Montréal, Montreal, Quebec Canada; 3 Centre de rhumatologie St-Louis, Sainte-Foy, Quebec Canada; 4 Groupe de recherche en Rhumatologie et Maladies Osseuses Inc., Sainte-Foy, Quebec Canada; 5 Centre de recherche musculo-squelettique, Trois-Rivières, Quebec Canada; 6 Service de rhumatologie, Centre hospitalier universitaire de Sherbrooke (CHUS), Sherbrooke, Quebec Canada; 7 ArthroLab Inc., Montreal, Quebec Canada; 8 StatSciences Inc., Notre-Dame de l’Île-Perrot, Quebec Canada

**Keywords:** Chondroitin sulfate, Symptomatic slow-acting drug in osteoarthritis, Osteoarthritis, Knee, Celecoxib

## Abstract

**Background:**

In osteoarthritis (OA) treatment, although chondroitin sulfate (CS) was found in a number of studies using radiography to have a structure-modifying effect, to date CS use is still under debate. A clinical study using quantitative magnetic resonance imaging (qMRI) is therefore of the utmost importance. Here we report data from a 24-month, randomised, double-blind, double-dummy, controlled, comparative exploratory study of knee OA. The primary endpoint was to determine the effect of CS 1200 mg/day versus celecoxib 200 mg/day on cartilage volume loss (CVL) in the lateral compartment over time as measured by qMRI. Secondary endpoints included assessment of the OA structural changes and signs and symptoms of OA.

**Methods:**

qMRI was performed at baseline and at 12 and 24 months. CVL, bone marrow lesion size, and synovial thickness were evaluated using qMRI. The primary statistical analysis was carried out on the modified intention-to-treat (mITT) population (*n* = 138) using chi-squared, Fisher’s exact, Wilcoxon Mann–Whitney, and Student’s *t* tests and analysis of covariance. Analyses were also conducted on the according-to-protocol (ATP; *n* = 120) population.

**Results:**

In the adjusted mITT analysis, compared with celecoxib treatment, patients treated with CS had a significant reduced CVL at 24 months in the medial compartment (celecoxib –8.1 % ± 4.2, CS –6.3 % ± 3.2; *p* = 0.018) and medial condyle (–7.7 % ± 4.7, –5.5 % ± 3.9; *p* = 0.008); no significant effect was seen in the lateral compartment. In the ATP population, CS reduced CVL in the medial compartment at 12 months (celecoxib –5.6 % ± 3.0, CS –4.5 % ± 2.6; *p* = 0.049) and 24 months (celecoxib –8.4 % ± 4.2, CS –6.6 % ± 3.3; *p* = 0.021), and in the medial condyle at 24 months (celocoxib –8.1 % ± 4.7, CS –5.7 % ± 4.0; *p* = 0.010). A trend towards a statistically reduced synovial thickness (celecoxib +17.96 ± 33.73 mm, CS –0.66 ± 22.72 mm; *p* = 0.076) in the medial suprapatellar bursa was observed in CS patients. Both groups experienced a marked reduction in the incidence of patients with joint swelling/effusion and in symptoms over time. Data showed similar good safety profiles including cardiovascular adverse events for both drugs.

**Conclusion:**

This study demonstrated, for the first time in a 2-year randomised controlled trial using qMRI, the superiority of CS over celecoxib at reducing CVL in knee OA patients.

**Trial registration:**

ClinicalTrials.gov NCT01354145. Registered 13 May 2011.

**Electronic supplementary material:**

The online version of this article (doi:10.1186/s13075-016-1149-0) contains supplementary material, which is available to authorized users.

## Background

Knee osteoarthritis (OA) is one of the most common musculoskeletal disorders, which causes joint pain, stiffness, and loss of function [1, 2]. Current treatment recommendations include a combination of non-pharmacological (e.g. education, exercise, weight loss) and pharmacological (e.g. acetaminophen, non-steroidal anti-inflammatory drugs (NSAIDs)) treatments, and some also suggest the use of symptomatic slow-acting drugs for osteoarthritis (SYSADOA) [3–5], which include chondroitin sulfate (CS) and glucosamine. However, the use of the latest line of treatment has not reached a general consensus among different guidelines [6, 7], due in part to the unavailability of prescription quality SYSADOAs indicated for use in OA that have been evaluated by the US Food and Drug Administration [6].

The goals of OA therapy are to decrease pain and maintain or improve joint function. Existing pharmacologic therapies for OA, namely NSAIDs and analgesics, help to reduce symptoms, but are only moderately effective and expose patients to potential significant toxicity (cardiovascular, hepatic, renal, and other adverse events (AEs)) and problems resulting from interactions with other medications. A recent systematic literature review suggests that paracetamol, especially at the upper end of standard analgesic doses, can also induce gastrointestinal events (i.e. gastroduodenal ulcers and complications such as upper gastrointestinal haemorrhages) and renal events (decrease in glomerular filtration rate) [8]. For this reason, attention has recently been focused on the investigation and development of new types of drugs and treatments that can improve the clinical symptoms of OA and show better safety profiles, such as symptomatic SYSADOAs. SYSADOAs such as CS are characterised by a slow onset of action and a global efficacy at reducing OA symptoms similar to that of NSAIDs, and also possess a carry-over effect. Furthermore, 6-month randomised controlled trials (RCTs) using similar grade preparations have shown that CS 1200 mg/day plus glucosamine 1500 mg/day can provide significant pain relief over placebo [9] and comparable pain relief with celecoxib [10] in a subgroup of patients with moderate-to-severe knee pain.

CS is a natural product extracted from animal cartilage (e.g. bovine trachea). A recent Cochrane review concluded that CS, alone or in combination with glucosamine, has a beneficial effect on pain and joint space narrowing (JSN) in patients with knee OA [11]. Of note, different sources of CS with variable composition and purity have been used in these studies, which may explain some heterogeneity of results [12]. RCTs using pharmaceutical-grade preparations of CS have shown that at 800 mg/day the treatment significantly reduced JSN as assessed using X-ray imaging [13–15], and decreased cartilage volume loss as evaluated by magnetic resonance imaging (MRI) in a pilot study [16]. In OA joint tissues, CS has been shown to modify the chondrocyte death process, to improve the anabolic/catabolic balance of the extracellular cartilage matrix, to reduce some pro-inflammatory and catabolic factors, and to reduce the resorptive properties of subchondral bone osteoblasts [17–21]. These findings support the mode of action of CS at reducing OA structural change progression.

The aim of the current study was to ascertain whether CS at 1200 mg/day, the maximum and most commonly used therapeutic dosage of CS alone or in combination with other drugs/agents such as glucosamine, over a period of 24 months would reduce cartilage volume loss, as assessed by MRI. Celecoxib was chosen as the comparator for many reasons. First, it was shown in an RCT using MRI that celecoxib and placebo have a non-different effect on the progression of cartilage volume loss in knee OA patients [22]. Moreover, celecoxib has an established efficacy in knee OA treatment and widespread use [23]. It is preferred over other NSAIDs due to its lower incidence of gastrointestinal side effects.

## Methods

### Study design

This 24-month randomised, double-blind, double-dummy controlled study (ClinicalTrials.gov NCT01354145) of patients with symptomatic knee OA and clinical synovitis aimed to explore the effect of CS and celecoxib on knee OA cartilage volume loss. The study was performed in Quebec, Canada. Patients were recruited by physicians in four private clinics and one outpatient clinic (Service de rhumatologie, Centre hospitalier universitaire de Sherbrooke (CHUS), Sherbrooke, QC, Canada).

### Patients

The study enrolled ambulatory men and women aged 40 years or older with primary symptomatic knee OA whose condition justified symptomatic treatment, as described previously [16]. They were diagnosed according to the clinical and radiological criteria of the American College of Rheumatology (ACR) [24], with clinical signs of synovitis (warmth, swelling, or effusion), a disease severity grade of 2–3 based on Kellgren–Lawrence radiographic scoring [25], a minimal medial joint space width of 2 mm on standing knee X-ray scan, and a visual analogue scale (VAS) pain index of at least 40 mm while walking. The X-ray inclusion criteria were selected not only to comply with ACR recommendations but also to avoid selection of patients with secondary OA and/or with too severe disease such as knee varus/valgus malalignment. Concomitant femoropatellar OA was not quantified by X-ray imaging. Participants were required to have no significant laboratory abnormalities. If both knees were affected by OA, the target knee with the most pronounced symptoms was selected if within the inclusion criteria. Full inclusion and exclusion criteria are presented in Additional file 1: Table S1.

### Treatments

Patients were assigned sequentially in a 1:1 ratio according to a predefined randomisation scheme using a mathematical algorithm and a randomisation list (Ropack Pharmaceutical Packaging, Montreal, QC, Canada). The list contained assignment of sequential numbers to one of the two treatment groups, pharmaceutical-grade CS (Bioibérica S.A., Barcelona, Spain) 1200 mg (three 400 mg capsules in the morning) or celecoxib (Pfizer Canada, Saint-Laurent, QC, Canada) 200 mg (one 200 mg capsule + two placebo capsules in the morning) for 24 months. All products were over-encapsulated to have the same appearance, size, and colour. The patients, site personnel, and sponsor were blinded to treatment.

Patients were not permitted to take other NSAIDs during the study (or during the week before randomisation), but could take acetaminophen (up to 3 g/day) with consumption interrupted 48 hours preceding evaluations.

The prior and concomitant treatment, blinding, treatment compliance, and study schedule are described in Additional file 1: Methods.

### Outcomes

The primary endpoint was percentage of cartilage volume loss over time in the lateral compartment (condyle and tibial plateau) of the target knee from baseline to 24 months. The decision for selecting this criterion was based on the findings of the pilot study [16] and those of other similar RCTs [26, 27]. The secondary endpoints included changes in percentage of cartilage volume loss in the medial compartment, synovitis severity (thickness of the synovial membrane), bone marrow lesion (BML) grade, and synovial fluid volume. The outcomes also included assessment on the VAS of knee pain, Western Ontario and McMaster Universities Osteoarthritis Index (WOMAC) scores, quality of life (QoL) Short Form-36 General Health (SF-36) scores, clinical evaluation (swelling, visual examination; effusion, bulge sign), analgesic consumption, and AEs, which were recorded during physical examinations at the baseline visit and follow-up visits at months 3, 6, 9, 12, 18, and 24. Safety outcomes included discontinuation of study treatment due to AEs, and changes in various laboratory measures and vital signs.

#### Knee MRI acquisitions

MRI was performed at baseline and at 12 and 24 months on 1.5 T scanners (Siemens, Erlangen, Germany; General Electric, Milwaukee, WI, USA) using a standard knee coil. The sequence acquisitions were as described previously for the cartilage [28] and synovial membrane [29].

The cartilage volume was measured by two experienced readers trained by musculoskeletal radiologists using the computer program Cartiscope™ (ArthroLab, Montreal, QC, Canada) as described previously [30, 31]. The readers were blinded to treatment and to MRI examination time points except for the baseline. The change in knee cartilage volume was obtained by subtracting the follow-up volume from the initial (baseline) volume. The reproducibility of the method has been demonstrated previously to be excellent: root mean square (RMS) coefficient of variation percentage (CV%) of 2.2 % for the global cartilage volume, 1.6 % for the medial compartment, and 2.6 % for the lateral compartment [30] and inter-reader and intra-reader intra-class correlation (ICC) of 0.94–0.99 [32]. In addition, the reproducibility of the reading is evaluated periodically using standard operating procedures (SOP; ArthroLab). The data show consistency of the method over time.

The extent of synovitis was assessed by measuring synovial thickness (mm) in four regions of interest (ROIs): the medial and lateral articular recess and the medial and lateral outer wall of the suprapatellar bursa [29]. The inter-reader and intra-reader correlation was excellent with ICC of 0.82–0.91 [29] and consistency of the method over time (SOP; ArthroLab). Of note, the measurement of the synovial membrane thickness according to this method relies on the presence of synovial fluid to localise the membrane in the different ROIs. An absence of synovial fluid, especially in the area of the medial suprapatellar bursa, thus accounts for missing values.

The synovial fluid was determined using a fully automated system as described previously [33]. Validation experiments revealed excellent coefficients of variation with a calibrated cylinder (1.4 %), sphere phantoms (0.8 %), manual quantification (*r* = 0.98, *p* < 0.0001), and comparison with direct aspiration (*r* = 0.88, *p* = 0.0008) [33].

BML were assessed in the same MRI sequences used for the cartilage [34]. The extent of the BML was evaluated in the global knee and each of the subregions using the following scale: 0, absence; 1, <25 %; 2, 25–50 %, 3, >50 % of the surface of the respective region regardless of the presence of additional smaller lesions. Reliability of the scoring system for BML changes was found to be excellent with ICC ranging from 0.88 to 0.93 [34] and consistency of the method over time (SOP; ArthroLab).

### Statistical analysis

The study populations were carefully defined in the statistical analysis plan (SAP). The safety population included all randomised patients who received at least one dose of study treatment (*n* = 194 at baseline). The modified intention-to-treat (mITT) population comprised all randomised patients who received at least one dose of study medication and for whom at least one post-baseline efficacy MRI measurement was available. The according-to-protocol (ATP) population included patients who fully complied with the study’s 24-month protocol and for whom all MRI evaluations were available. Data were entered into a computerised database using a blinded double-entry procedure, after which descriptive statistics for patient characteristics were tabulated. Before locking the database, the following actions were performed under conditions blinded for the type of intervention: the database was cleaned, all queries were resolved, a review meeting regarding blinded data was conducted, protocol deviations were identified, the SAP was developed, and signed approval was obtained. Following the final database lock, the statistical analysis was performed by an independent expert biostatistics firm (Inferential, Paris, France).

Descriptive variables at baseline are presented as number (percentage) or mean ± standard deviation (SD). Differences between the two treatment groups were assessed using a Kruskal–Wallis test or Student’s *t* test for quantitative variables, and a chi-squared test or Fisher’s exact test for categorical variables. Analgesic consumption was tallied and compared between the two groups. The primary efficacy outcome measure for structure modification was percentage cartilage volume loss in the lateral compartment of the target knee after 24 months of enrolment for the mITT population who had at least one post-baseline MRI measurement while using, as specified in the SAP, the imputation method of the last observation carried forward (mITT-LOCF). Because of the difference at baseline in body mass index (BMI) values between the two treatment groups (Table 1), an analysis of covariance (ANCOVA) including BMI as a covariate was performed on the mITT-LOCF population. In order to provide an additional measure of treatment efficacy, the ATP population (i.e. patients who completed the study according to the 24-month protocol) was also assessed using the ANCOVA method. Additionally, the data from the mITT and ATP populations were also analysed using a generalised linear mixed-model analysis. Secondary efficacy analyses of structural changes were done using the same methodology. Comparison of symptom changes using the WOMAC questionnaire, VAS pain, QoL SF-36, knee swelling and effusion, and safety were assessed on all available data as specified in the SAP.

**Table 1:** Randomised patient characteristics at baseline

	Chondroitin sulfate (*n* = 97)	Celecoxib (*n* = 97)
Female	53 (54.6)	61 (62.9)
Age (years)	61.4 ± 9.3	61.3 ± 8.5
Body mass index (kg/m^2^)	30.1 ± 5.8	32.3 ± 5.8
At least one previous medical condition	86 (88.7)	86 (88.7)
At least one active medical condition	96 (99.0)	97 (100.0)
Cartilage volume (mm^3^)		
Lateral compartment	4613 ± 1755	4405 ± 1157
Condyle	2621 ± 1073	2476 ± 716
Plateau	1993 ± 799	1930 ± 540
Medial compartment	4515 ± 1537	4255 ± 1217
Condyle	2807 ± 971	2646 ± 779
Plateau	1708 ± 623	1609 ± 489
Synovial membrane thickness (mm)	1.0 ± 0.2	1.0 ± 0.2
BML score (global knee)^a^	2.6 ± 3.0	2.7 ± 2.5
Synovial fluid volume (ml)	13.8 ± 14.6	10.8 ± 10.9
Joint swelling and effusion		
Swelling	76 (78.4)	78 (80.4)
Effusion	76 (78.4)	71 (73.2)
Both	59 (60.8)	55 (56.7)
Pain VAS (mm)^a^	62.42 ± 15.51	59.26 ± 18.10
WOMAC^a^		
Total score (0–240)	124.9 ± 38.1	126.7 ± 43.9
Pain score (0–50)	25.6 ± 8.1	25.5 ± 9.0
Stiffness score (0–20)	10.8 ± 3.7	11.7 ± 4.3
Physical function score (0–170)	88.5 ± 28.7	89.5 ± 32.5
Quality of life (SF-36)^a^		
Physical component summary	35.4 ± 7.8	35.7 ± 8.1
Mental component summary	51.7 ± 9.5	52.7 ± 11.0

Data shown as number of patients (%) or mean ± standard deviation

^a^Data were not available for two patients in the chondroitin sulfate group

*BML* bone marrow lesion, *SF-36* Short Form-36, *VAS* visual analogue scale, *WOMAC* Western Ontario and McMaster Universities Osteoarthritis Index

No sample size estimation was carried out because this was an exploratory study. Statistical tests were two-sided and significance reached at *p* < 0.05. No statistical adjustments were made for multiple comparisons while analysing secondary outcomes. Statistical analyses were performed using SAS® software version 9.3 (SAS Institute, Cary, NC, USA).

## Results

### Patients

**Fig. 1:**
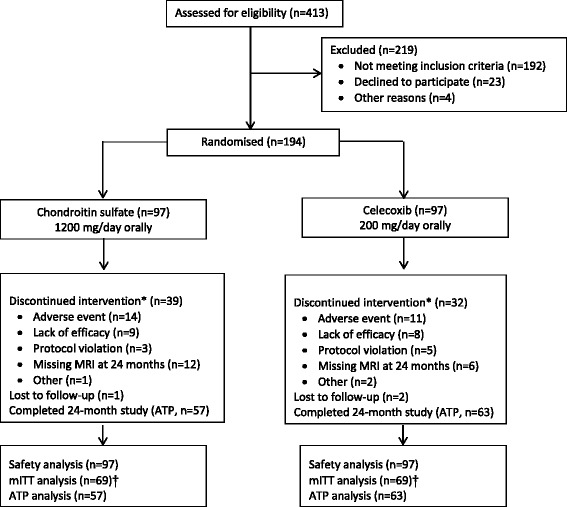
Patient disposition. *Primary reasons for discontinuation. ^†^mITT includes ATP patients plus those with MRI at 12 months but with MRI missing at final visit. *ATP* according-to-protocol, *mITT* modified intention-to-treat, *MRI* magnetic resonance imaging

The study was conducted from 21 June 2011 to 10 September 2014. A total of 194 patients were randomised to CS or celecoxib. The study populations used for analyses are as described in Fig. 1.

No differences were found in the baseline characteristics of the patient populations (Table 1) with the exception of BMI; although values for both groups were in the obesity category (≥30 kg/m^2^), a higher value was found in the celecoxib group.

No significant difference between the treatment groups was found for previous or concomitant medications (Additional file 1: Table S2).

### Efficacy

#### Cartilage volume

In the adjusted mITT-LOCF analysis (*n* = 138), the percentage cartilage volume loss in the lateral compartment (primary endpoint) was not different in the CS (*n* = 69) and celecoxib (*n* = 69) groups at 24 months (Table 2). In the medial compartment and medial condyle, the cartilage volume loss was significantly less in the CS group at 24 months (*p* = 0.018 and *p* = 0.008, respectively) compared with celecoxib. The results from the generalised linear mixed-model analysis at 24 months showed *p* = 0.038 for the medial compartment and *p* = 0.015 for the medial condyle. However, the results of the adjusted analysis for the ATP population (*n* = 120; Table 2) showed a significant reduction in cartilage volume loss in CS-treated patients at 12 months for the medial compartment (*p* = 0.049) and at 24 months for the medial compartment and medial condyle (*p* = 0.021 and *p* = 0.010, respectively). The results from the generalised linear mixed-model analysis showed *p* = 0.043 and *p* = 0.035 for the medial compartment at 12 months and 24 months, respectively, and *p* = 0.015 for the medial condyle at 24 months.

**Table 2:** Changes in MRI parameters

mITT-LOCF population	24 months					
	Chondroitin sulfate	Celecoxib	*p* value^a^			
	(*n* = 69)	(*n* = 69)				
Cartilage volume loss (%)						
Lateral compartment	–4.1 ± 3.1	–4.4 ± 3.0	0.814			
Condyle	–3.0 ± 3.1	–4.1 ± 3.7	0.144			
Plateau	–5.8 ± 4.7	–4.8 ± 3.5	0.182			
Medial compartment	–6.3 ± 3.2	–8.1 ± 4.2	**0.018**			
Condyle	–5.5 ± 3.9	–7.7 ± 4.7	**0.008**			
Plateau	–7.6 ± 4.0	–8.6 ± 4.8	0.276			
Synovial membrane thickness^b^ (mm)	0.13 ± 0.25	0.14 ± 0.24	0.948			
Synovial fluid volume (ml)	–2.6 ± 14.5	–2.0 ± 11.8	0.776			
BML score^b^	1.1 ± 1.7	0.8 ± 1.7	0.322			
ATP population	12 months			24 months		
	Chondroitin sulfate	Celecoxib	*p* value^a^	Chondroitin sulfate	Celecoxib	*p* value^a^
	(*n* = 57)^c^	(*n* = 63)		(*n* = 57)^c^	(*n* = 63)	
Cartilage volume loss (%)						
Lateral compartment	–3.4 ± 2.7	–3.3 ± 2.5	0.932	–4.6 ± 3.0	–4.4 ± 2.8	0.753
Condyle	–2.5 ± 3.2	–3.0 ± 3.0	0.234	–3.4 ± 3.0	–4.2 ± 3.6	0.316
Plateau	–4.6 ± 3.5	–3.8 ± 3.1	0.194	–6.3 ± 4.6	–4.8 ± 3.5	0.081
Medial compartment	–4.5 ± 2.6	–5.6 ± 3.0	**0.049**	–6.6 ± 3.3	–8.4 ± 4.2	**0.021**
Condyle	–3.8 ± 3.2	–5.0 ± 3.8	0.100	–5.7 ± 4.0	–8.1 ± 4.7	**0.010**
Plateau	–5.5 ± 3.0	–6.6 ± 3.8	0.155	–8.0 ± 4.2	–9.0 ± 4.8	0.334
Synovial membrane thickness^b^ (mm)	0.03 ± 0.18	0.06 ± 0.18	0.579	0.15 ± 0.26	0.15 ± 0.24	0.731
Synovial fluid volume (ml)	–5.5 ± 12.6	–1.8 ± 9.2	0.326	–3.9 ± 15.4	–2.1 ± 12.2	0.945
BML score^b^	0.7 ± 1.4	0.3 ± 1.2	**0.051**	1.1 ± 1.7	0.8 ± 1.7	0.322

Data are mean ± standard deviation

^a^Analysis of covariance; model includes BMI as covariate; bold indicates statistical significance

^b^Global knee

^c^Among the 58 patients in the chondroitin sulfate group who completed the study, one patient did not complete the MRI at 24 months

*ATP* according-to-protocol, *BMI* body mass index, *BML* bone marrow lesion, *LOCF* last observation carried forward, *mITT* modified intention-to-treat, *MRI* magnetic resonance imaging

#### Synovial membrane

The assessment of the mean synovial thickness in the global knee (four ROIs together) was not different between treatment groups at baseline (Table 1). The change in synovial membrane thickness between the two therapeutic groups was not different at any time point in both mITT (*n* = 138) and ATP (*n* = 120) population analyses (Table 2). However, in post-hoc analysis, we also examined the changes in synovial thickness in one of the four ROIs, the medial suprapatellar bursa (*n* = 50; Table 3). Because such measurement is reliant on the presence of synovial fluid to localise the membrane, the absence of synovial fluid accounts for missing values. Data showed, in patients for whom the assessment of thickness was possible at baseline and at 24 months, a numerical trend (*p* = 0.076) towards a decrease at 24 months in the CS group versus the celecoxib group. This is associated with a significant decrease (*p* = 0.045) in the cartilage volume loss in the medial compartment. Moreover, when analysed based on whether patients had experienced a decrease or increase in synovial membrane thickness from baseline to 24 months (Table 3), data showed that patients treated with CS had a significantly lesser increase (*p* = 0.030) in synovial membrane thickness compared with those in the celecoxib group. The patients in the CS group who experienced a decrease in synovial membrane thickness had significantly less cartilage volume loss (*p* = 0.036) than those in the celecoxib group.

**Table 3:** Changes at 24 months in synovial membrane thickness and cartilage volume

				Decrease^a^			Increase^a^		
	Chondroitin sulfate (*n* = 26)^b^	Celecoxib (*n* = 24)^b^	*p* value^c^	Chondroitin sulfate (*n* = 12)	Celecoxib (*n* = 8)	*p* value^c^	Chondroitin sulfate (*n* = 12)	Celecoxib (*n* = 14)	*p* value^c^
Medial suprapatellar bursa									
Synovial membrane thickness loss (mm)	–0.66 ± 22.72	+17.96 ± 33.73	0.076	–0.23 ± 0.27	–0.14 ± 0.21	0.446	+0.13 ± 0.09	+0.25 ± 0.17	**0.030**
Medial compartment									
Cartilage volume loss (%)	–6.8 ± 3.5	–9.4 ± 4.7	**0.045**	–7.5 ± 3.7	–11.3 ± 3.5	**0.036**	–6.4 ± 3.4	–7.8 ± 4.8	0.404

Data are mean ± standard deviation

^a^Data from patients who presented a decrease or an increase in synovial membrane thickness at 24 months

^b^Synovial membrane thickness measurement is reliant on the presence of synovial fluid to localise the membrane; absence of synovial fluid accounts for missing values

^c^Student’s *t* test or Wilcoxon Mann–Whitney test, bold indicates statistical significance

#### Synovial fluid volume and BML score

Analyses showed no significant differences between the two treatment groups in baseline values (randomised patients, *n* = 194; Table 1) or in the changes (mITT, *n* = 138; ATP, *n* = 120; Table 2) in synovial fluid volume. For BML, the only significance was found in the changes at 12 months (*p* = 0.051) in the ATP population.

#### Joint effusion/swelling

The incidence of joint effusion and/or swelling decreased similarly in both groups during the study in the randomised patient population (all available data, baseline, *n* = 194; Additional file 1: Figure S1). A marked reduction in the incidence (%) of patients with joint swelling plus effusion was observed in both CS and celecoxib groups (42 % and 29 %, respectively) at 24 months.

#### Symptoms and function

**Fig. 2:**
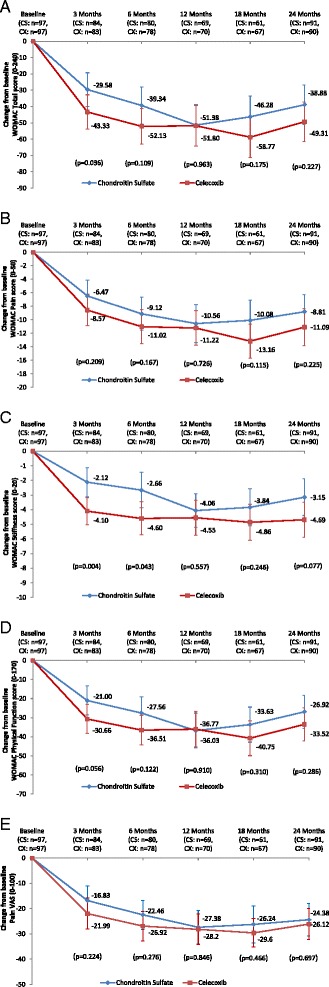
Change in symptoms and function from baseline based on available data. WOMAC (**a**) total, (**b**) pain, (**c**) stiffness, and (**d**) function subscales, and (**e**) VAS, by visit. Data are mean of the relative change (%) from baseline. Statistical analyses comparing chondroitin sulfate and celecoxib were performed using Student’s *t* test or the Kruskal–Wallis test. *CS* chondroitin sulfate, *CX* celecoxib, *WOMAC*, Western Ontario and McMaster Universities Osteoarthritis Index, *VAS* visual analogue scale

Both therapeutic groups experienced a reduction in disease symptoms over time (all available data; baseline *n* = 194) (Fig. 2). The decrease in WOMAC scores (Fig. 2a–d) was slightly more pronounced in the celecoxib group compared with the CS group, mostly at earlier time points (months 3 and 6), but statistical significance was never reached except for the WOMAC total at month 3 (Fig. 2a) and stiffness at months 3 and 6 (Fig. 2c). At 24 months, the reduction in WOMAC pain (Fig. 2b) was 36 % for the CS group and 42 % for the celecoxib group. The level of pain on VAS decreased over time in both treatment groups (Fig. 2e). The reduction at 24 months was 38 % and 43 % for the CS and celecoxib groups, respectively (Fig. 2e).

With regards to the QoL SF-36 physical and mental component scores, an improvement in QoL was found for both groups without significant differences between them (data not shown).

The overall daily consumption of rescue analgesic (acetaminophen) was not different between the CS and celecoxib groups (584 versus 472 mg/day).

### Safety

Study durations (mean ± SD) were 581 ± 254 and 579 ± 262 days in the CS and celecoxib groups, respectively (*n* = 194), and treatment durations were 543 ± 260 and 564 ± 263 days, respectively.

A total of 78 patients (80.4 %) in the CS group and 79 patients (81.4 %) in the celecoxib group reported at least one AE during the study. The most frequent AEs (Table 4) at the system organ class (SOC) level were musculoskeletal and connective tissue disorders, infections and infestations, and gastrointestinal disorders. The most common AEs by preferred term were nasopharyngitis, arthralgia, dyspepsia, headache, and back pain. Significant differences between the two groups were seen only for back pain and skin and subcutaneous tissue disorders (Table 4). The vascular disorders described in the table were hypertension (CS, *n* = 4; celecoxib, *n* = 3) and hot flush (CS, *n* = 1). Most emergent AEs were mild to moderate with no significant difference between treatment groups (Table 5). The overall frequency of patients with emergent AEs considered possibly or probably related to study treatment, including cardiovascular events (mainly hypertension; CS, *n* = 4, celecoxib, *n* = 4), was similar in the two groups. Findings regarding the AEs leading to study withdrawal were also similar in the two therapeutic groups. No death occurred during the study. Ten patients in the CS group versus six in the celecoxib group experienced at least one serious adverse event (SAE). One SAE (iron-deficiency anaemia) was considered by the investigator to be related to treatment in one patient in the CS group and two SAEs (pneumonia and pulmonary embolism) in one patient in the celecoxib group.

**Table 4:** Frequency of patients with at least one emergent adverse event (occurring in ≥5 % of patients): safety population

	Chondroitin sulfate (*n* = 97)		Celecoxib (*n* = 97)		*p* value^a^
	Events	*n* (%)	Events	*n* (%)	
Musculoskeletal and connective tissue disorders	72	37 (38.1)	59	38 (39.2)	>0.999
Arthralgia	19	14 (14.4)	14	11 (11.3)	0.669
Back pain	17	16 (16.5)	5	5 (5.2)	**0.019**
Pain in extremity	6	3 (3.1)	8	7 (7.2)	0.331
Infections and infestations	66	34 (35.1)	61	32 (33.0)	0.880
Nasopharyngitis	28	19 (19.6)	18	15 (15.5)	0.572
Gastrointestinal disorders	37	27 (27.8)	44	29 (29.9)	0.874
Dyspepsia	12	12 (12.4)	12	12 (12.4)	1.000
Gastroesophageal reflux disease	5	5 (5.2)	5	5 (5.2)	1.000
Nervous system disorders	21	16 (16.5)	22	21 (21.6)	0.465
Headache	15	12 (12.4)	10	10 (10.3)	0.821
Respiratory, thoracic, and mediastinal disorders	19	15 (15.5)	20	14 (14.4)	>0.999
Sinusitis	5	5 (5.2)	6	6 (6.2)	>0.999
Injury, poisoning, and procedural complications	18	14 (14.4)	18	13 (13.4)	>0.999
General disorders and administration site conditions	12	11 (11.3)	12	11 (11.3)	1.000
Skin and subcutaneous tissue disorders	3	3 (3.1)	11	11 (11.3)	**0.049**
Psychiatric disorders	4	4 (4.1)	9	9 (9.3)	0.250
Investigations	6	6 (6.2)	6	6 (6.2)	1.000
Surgical and medical procedures	8	5 (5.2)	8	6 (6.2)	>0.999
Vascular disorders^b^	7	6 (6.2)	5	4 (4.1)	0.747

Data shown are number of events or patients (%)

^a^Chi-squared test or Fisher’s exact test for proportions (%), bold indicates statistical significance

^b^Vascular disorders include mainly hypertension, varicose veins, and ulcers

**Table 5:** Summary of emergent adverse events: safety population

	Chondroitin sulfate (*n* = 97)		Celecoxib (*n* = 97)		*p* value^a^
	Events	*n* (%)	Events	*n* (%)	
At least one AE	305	78 (80.4)	299	77 (79.4)	>0.999
Intensity					
Mild	203	61 (62.9)	215	61 (62.9)	1.000
Moderate	89	47 (48.5)	68	41 (42.3)	0.471
Severe	13	9 (9.3)	16	10 (10.3)	>0.999
SAE	16	10 (10.3)	9	6 (6.2)	0.435
Relationship to study treatment					
Not related	246	67 (69.1)	241	68 (70.1)	>0.999
Uncertain	20	14 (14.4)	23	13 (13.4)	>0.999
Related^b^	39	27 (27.8)	35	24 (24.7)	0.745
AE that led to study withdrawal	13	13 (13.4)	12	11 (11.3)	0.828
Ongoing^c^	95	50 (51.5)	100	50 (51.5)	1.000

^a^Chi-squared test for proportions (%)

^b^Relationship considered possible, probable, or very probable

^c^Not resolved/recovered at the end of the study

*AE* adverse event, *SAE* severe adverse event

## Discussion

In this 24-month RCT, although we found no statistically significant reduction in cartilage volume loss by CS treatment in the lateral compartment, which was the primary outcome of the study, CS at 1200 mg/day was found to have a beneficial effect versus celecoxib 200 mg/day on cartilage volume loss in the medial compartment in knee OA patients. Moreover, CS, to a greater extent than celecoxib, induced a lesser increase in synovial thickness in the medial suprapatellar bursa that was associated with a decrease in cartilage volume loss in the medial compartment. There was also no evidence of superiority of one treatment over the other at improving disease symptoms except joint stiffness in the early phase of the study, which was greater in celecoxib patients. These findings provide new information about the potential benefit of long-term treatment with CS in knee OA patients.

The reduction in cartilage volume loss found with CS in the current study is in line with several other RCTs [13–16] and meta-analyses and reviews [11, 35–38], in which MRI (cartilage volume) or X-ray imaging (JSN) were used to assess disease progression, a number of which used a placebo as a comparator [13–15], including a pilot study using MRI [16]. Of note, the results of a recent study in knee OA patients [39] reported a significant reduction in cartilage volume loss with CS plus glucosamine, but not with CS alone, compared with patients treated with placebo. The results of the latter [39], which used X-ray imaging and JSN to assess the disease-modifying osteoarthritis drug (DMOAD) effect, could possibly be explained by the fact that it was underpowered and of too short a duration for such imaging technology to allow an accurate assessment of possible changes related to treatment. Some other meta-analyses [40–42] have reported no beneficial effect of CS on JSN. Such findings can be at least partly explained by the heterogeneity between results of the studies included in the analyses, which may have been related, to some extent, to the use of non-pharmaceutical-grade CS as the therapeutic agent in some trials [12, 43, 44].

The change in cartilage volume in the lateral compartment was chosen as the primary endpoint based mainly on the results of the pilot study which reported significantly less cartilage volume loss in the lateral compartment with CS versus placebo [16], as well as two other knee OA RCTs using MRI [26, 27]. Here, the protective effect was found only in the medial compartment. This discrepancy could probably be explained by many factors including the difference in disease severity between the patients in each study. Patients in the current study had less severe disease than those in the pilot study [16], as evidenced by higher knee cartilage volume, particularly in the medial compartment. Because knee OA structural damage (cartilage volume loss) in such patients is predominantly seen in the medial compartment, disease progression in the lateral compartment may not have been sufficient for CS to demonstrate a significant therapeutic effect, as shown in a recent report [45]. Moreover, the duration of treatment in the pilot study of only 12 months and a lower dosage of CS (800 mg/day) may have precluded the finding of a positive effect in the medial compartment. Nevertheless, we believe that the present findings are of great importance from many aspects. First, they are very clinically relevant, as a recent consensus of experts recommends cartilage volume loss in the medial compartment as a clinical trial outcome [46]. The experts agree that it can predict knee replacement, is sensitive to change, and predicts outcome in a continuous manner. This is quite interesting in the context of the present study, because CS treatment was reported previously to have a beneficial effect on the need for total knee replacement in a 4-year follow-up study in knee OA [47]. A significant number of the patients (*n* = 134) who were randomised in the study are now being followed for a period of 4 years after study completion to evaluate the cumulative incidence of total knee replacement to ascertain whether the finding of our previous study [47] of the sparing effect of CS treatment on knee replacement could be confirmed. The results of the present study are also most helpful with regard to the selection of primary endpoints in future DMOAD RCTs. The selection of a very specific region of the knee in OA trials may not be the most optimal approach, as demonstrated in this study and in another recent RCT report [26]. This obviously needs to be examined more closely as results from additional RCTs become available.

The finding of a reduction in synovitis in the medial compartment by CS treatment in certain patients is interesting. It provides a hypothesis about the mechanism by which the effect of CS on cartilage volume loss is mediated. It also supports the findings of the pilot study regarding the effect of CS on synovitis [16].

The finding that CS had a beneficial effect comparable with that of celecoxib on pain, stiffness, physical function, and QoL is in line with a recent randomised trial in which CS in combination with glucosamine provided non-inferior pain relief to celecoxib [10] and supports findings from previous trials [13, 15, 48] and meta-analyses [11, 42, 49] that have also reported a significant beneficial effect of CS (with or without glucosamine) on pain. Of note is that not all clinical studies with CS have shown a significant effect on pain [14, 39], while others found a significant effect only in patients with moderate to severe pain [9]. Differences among the patient populations, product formulations that were studied, and follow-up times between studies may explain, to some extent, those discrepancies.

Regarding safety, this study confirmed the good tolerability of both treatments for up to 24 months, with no new safety issue, including cardiovascular events, arising for either treatment. These findings are similar to those observed in the MOVES study [10], which compared the symptomatic efficacy of CS plus glucosamine hydrochloride versus celecoxib in knee OA patients. It should, however, be taken into consideration that the patients included in this study and in the MOVES study [10] were those with low-risk cardiovascular and gastrointestinal diseases, which could have impacted the safety results.

As with all studies, the present study has some limitations. The relatively small sample size of this exploratory study and less severe disease severity in the patient population may have contributed to an underestimation of the effects of CS in the lateral compartment. Although patients with knee malalignment were excluded, such angulation was not assessed per se as an outcome variable and may be associated with the knee compartment in which cartilage volume loss could progress despite therapy. Additionally, although there is a well-known placebo effect in OA [50], we did not include a placebo group because it was felt that this would have been unethical, particularly in a 24-month study, and CS has already been shown to have a beneficial effect on JSN and/or pain in a number of placebo-controlled studies in knee OA [13–16, 48]. Similarly, celecoxib has proven pain efficacy over placebo in OA in several studies [51–53]. Moreover, celecoxib was found to have a neutral effect similar to placebo on cartilage volume loss in knee OA patients [16]. The interruption period for acetaminophen before evaluations was sufficient to have no effect on the studied pain outcomes.

## Conclusion

This trial demonstrated, for the first time in a 24-month RCT using MRI, the superiority of CS over celecoxib at reducing the long-term progression of knee OA cartilage volume loss. Moreover, both drugs were found effective at reducing the symptoms of OA over the entire duration of the 24-month study, with no superiority of one over the other. These findings are interesting with regard to the potential usefulness of CS for long-term management of knee OA. A definitive study, however, will be required if one wishes to fully confirm the findings.

## Additional file

Additional file 1: Includes methods (prior and concomitant treatment, blinding, treatment compliance, study schedule), **Table S1.** presenting inclusion and exclusion criteria, **Table S2.** presenting previous or concomitant medications, and **Figure S1.** showing joint effusion and swelling. (DOCX 60 kb)

## Abbreviations

ACR: American College of Rheumatology
AE: Adverse event
ANCOVA: Analysis of covariance
ATP: According-to-protocol
BMI: Body mass index
BML: Bone marrow lesion
DMOAD: Disease-modifying osteoarthritis drug
CS: Chondroitin sulfate
CV%: Coefficient of variation percentage
JSN: Joint space narrowing
LOCF: Last observation carried forward
mITT: Modified intention-to-treat
MRI: Magnetic resonance imaging
NSAID: Non-steroidal anti-inflammatory drug
OA: Osteoarthritis
qMRI: Quantitative magnetic resonance imaging
QoL: Quality of life
RCT: Randomised controlled trial
RMS: Root mean square
ROI: Region of interest
SAE: Serious adverse event
SD: Standard deviation
SYSADOA: Symptomatic slow-acting drug for osteoarthritis
VAS: Visual analogue scale
WOMAC: Western Ontario and McMaster Universities Osteoarthritis Index
