# Supplementary material for: Chondroitin sulfate efficacy versus celecoxib on knee osteoarthritis structural changes using magnetic resonance imaging: a 2-year multicentre exploratory study
Source: Arthritis Res Ther. 2016 Nov 3;18:256. doi: 10.1186/s13075-016-1149-0 (PMC5094139; doi:10.1186/s13075-016-1149-0)
Supplement: Additional file 1: — Includes methods (prior and concomitant treatment, blinding, treatment compliance, study schedule), Table S1. presenting inclusion and exclusion criteria, Table S2. presenting previous or concomitant medications, and Figure S1. showing joint effusion and swelling. (DOCX 60 kb) [file 13075_2016_1149_MOESM1_ESM.docx]

**Additional file 1**

# CHONDROITIN SULFATE EFFICACY VERSUS CELECOXIB ON KNEE OSTEOARTHRITIS STRUCTURAL CHANGES USING MAGNETIC RESONANCE IMAGING: A TWO-YEAR MULTICENTRE EXPLORATORY STUDY

Pelletier JP, Raynauld JP, Beaulieu A, Bessette L, Morin F, de Brum-Fernandes AJ, Delorme P, Dorais M, Paiement P, Abram F, Martel-Pelletier J

**Methods**

**Prior and concomitant treatment**

Treatment with medication for osteoporosis at baseline was continued in an unmodified manner for the entire duration of the study if so wished. The medications and other treatments in use for intercurrent illnesses were recorded at the baseline visit and during the study whenever these treatments were modified.

**Blinding**

The investigators, subjects and sponsors were blinded to the allocated treatment through central randomisation. Sealed coded tamper-proof envelopes, specifying the treatment group for each study drug kit number, were provided to each centre. The envelopes were to be opened only in the event of an emergency.

**Treatment compliance**

The number of capsules of the study drug taken was calculated from the drug dispensation log. The compliance index was calculated as the percentage of the rated drug doses taken between the date of dispensation and the date of return of the study drugs. To remain in the study, a global compliance index of ≥75% was mandatory.

**Study schedule**

Patients had a screening visit 7-14 days prior to baseline MRI including physical examination for joint swelling or effusion, X-ray, and blood samples for routine laboratory tests to assess eligibility. A follow-up physical examination was performed at 3, 6, 12, 18 and 24 months.

**Table S1.** Inclusion and exclusion criteria

## Inclusion criteria

- Males or females aged ≥40 years
- Followed in an ambulatory clinic
- Presented primary osteoarthritis of the knee according to ACR criteria with signs of synovitis (warmth, swelling or effusion)
- Osteoarthritis of radiological stages 2 and 3 according to Kellgren-Lawrence
- Minimum JSW 2 mm in the medial tibiofemoral compartment on standing knee X-ray
- Knee pain for ≥1 month of the 3 months preceding the study
- Walking pain VAS ≥40 mm
- No clinical or significant laboratory abnormalities
- Female patients of childbearing potential must have had a negative pregnancy test at screening and agreed to use an acceptable method of birth control for the duration of the study
- Agreement to sign the Informed Consent Form prior to any study-related activities after having been clearly informed of its methods and constraints
- Not taking part in another clinical trial
- Agreement to respect the protocol by attending the visits related to the study

## Exclusion criteria related to patient characteristics

- Known allergy to chondroitin sulfate, hypersensitivity to celecoxib, demonstrated allergic-type reactions to sulphonamides, experienced asthma, urticaria or allergic-type reactions after taking sulphonamides, ASA, lactose, or NSAIDs
- Active malignancy of any type or history of a malignancy within the last 5 years other than basal cell carcinoma
- Increased risk for prostate cancer, with prostate cancer, or with a history of prostate cancer within the last 5 years
- Other bone and articular diseases (antecedents and/or current signs) such as chondrocalcinosis, Paget’s disease of the ipsilateral limb to the target knee, rheumatoid arthritis, aseptic osteonecrosis, gout, septic arthritis, ochronosis, acromegaly, haemochromatosis, Wilson’s disease, osteochondromatosis seronegative spondylo-arthropathy, mixed connective tissue disease, collagen vascular disease, psoriasis, inflammatory bowel disease
- Isolated knee lateral compartment osteoarthritis defined by joint space loss in the lateral compartment only
- Class IV functional capacity using ARA criteria
- Surgery in any lower limb or arthroscopy, aspiration, or lavage in any lower limb joint within 180 days of baseline
- Meniscal surgery on the study knee
- Total knee replacement in the contralateral knee within 6 months prior to screening and throughout the study. Patients who underwent a total knee replacement in the contralateral knee during the trial were considered withdrawn early, had to undergo an exit MRI evaluation, and a health professional had to fill out the End of Study Form
- Comorbid conditions that restrict knee function
- History of heart attack or stroke, or serious chest pain related to heart disease, or serious diseases of the heart such as congestive heart failure
- High risk of cardiovascular events, according to the AHA assessment of cardiovascular risk tables
- Any significant diseases or conditions, including emotional or psychiatric disorders and substance abuse that, in the opinion of the investigator, were likely to alter the course of osteoarthritis or the patient’s ability to complete the study
- Any active acute or chronic infections requiring antimicrobial therapy, or serious viral (e.g., hepatitis, herpes zoster, HIV positivity) or fungal infections
- History of recurrent upper gastrointestinal ulceration or active inflammatory bowel disease (e.g., Crohn’s disease or ulcerative colitis), a significant coagulation defect, or any other condition, which in the investigator’s opinion might have precluded the chronic use of celecoxib. Patients may have, at the investigator’s discretion, taken a proton pump inhibitor or antacids daily as required
- Diagnosed as having or having been treated for oesophageal, gastric, pyloric channel, or duodenal ulceration within 30 days prior to receiving the first dose of study medication
- Chronic liver or kidney disease, as defined by AST or ALT >2.0×ULN or blood urea nitrogen or serum creatinine >2.0×ULN at screening (the protocol was amended from >1.0×ULN for both)
- History of intolerance to acetaminophen, opioids, or opioid combinations such that it was felt that an adequate non-anti-inflammatory rescue analgesic regimen could not be safely prescribed
- History of alcohol or substance abuse
- Receipt of any investigational drug within 30 days or five half-lives (whichever is greater) prior to baseline
- Planned surgery during the trial
- Female patients who were breastfeeding
- Inability to take part in the total duration of the study and attend the visits
- Inability to give informed consent
- Lack of respect of the acetaminophen washout period of 48 hours and/or the NSAID washout period of 1 week before baseline

## Exclusion criteria related to treatment

- Use of corticosteroids (oral, injectable; exception of intra-articular/soft tissue injection at the exclusion of the target knee), indomethacin, tramadol, codeine, empracet, therapeutic dose of glucosamine or chondroitin sulfate during the 12 weeks preceding inclusion
- Use of hyaluronic acid (intra-articular target knee) during the 26 weeks preceding inclusion
- Use of natural health products (e.g., capsaicin, boswellia, willow bark) and creams and analgesic gels (e.g., camphor and alcohol-based gels) during 1 week preceding baseline
- Use of natural health products susceptible to increase the risk of bleeding (e.g., garlic, dong quai, etc.) during 1 week preceding baseline
- Receipt of radioactive synovectomy (target knee) during the 12 weeks preceding inclusion
- Patients who were receiving an NSAID and did not want to stop during the study
- If treatment of osteoporosis (bisphosphonates, SERMS, THS) was necessary, it had to be continued, unmodified, for the entire duration of the study
- Use of compounds containing non-approved agents for arthritis or agents claiming to possess disease/structure-modifying properties in the 14 days prior to baseline
- Use of medications with metalloproteinase-inhibitory properties (e.g., tetracycline or structurally related compounds) within 28 days prior to baseline
- Requirement of acetaminophen >3000 mg/day on a regular basis
- Use of lithium carbonate, phenytoin, or anticoagulants (with the exception of ASA up to a maximum daily dose of 325 mg)
- Chondrocyte transplants in any lower extremity joint
- Use of oral or topical cyclooxygenase inhibitors
- Use of calcitonin
- Use of immunosuppressive drugs

## Exclusion criteria related to MRI

- Contraindication to MRI examination
- If baseline cartilage volume could not be calculated from the MRI due to: advanced osteoarthritis disease, the presence of large fat knee pads, or any other technical reason
- Study knee did not enter the MRI magnet
- Abnormal baseline findings and/or any other condition which, in the investigator’s opinion, might have increased the risk to the patient or decreased the chance of obtaining satisfactory data through MRI to achieve the objectives of the study

AHA, American Heart Association; ALT, alanine aminotransferase; ARA, American Rheumatism Association; ASA, acetyl salicylic acid; AST, aspartate aminotransferase; HIV, human immunodeficiency virus; JSW, joint space width; MRI, magnetic resonance imaging; NSAID, non-steroidal anti-inflammatory drug; SERM, selective oestrogen receptor modulator; THS, thyroid-stimulating hormone; ULN, upper limit of normal.

**Table S2.** Previous medications reported in ≥5% of patients and concomitant medications reported in ≥10% of patients overall

|  | **Chondroitin**  **Sulfate**  **(n=97)** | **Celecoxib**  **(n=97)** | **p-value*** |
| --- | --- | --- | --- |
| Previous medications | | | |
| Coxibs | 7 (7.2) | 13 (13.4) | 0.237 |
| Anilides | 6 (6.2) | 9 (9.3) | 0.592 |
| Propionic acid derivatives | 8 (8.2) | 5 (5.2) | 0.568 |
| Acetic acid derivatives and related substances | 7 (7.2) | 4 (4.1) | 0.537 |
| Anti-inflammatory preparations, non-steroids for topical use | 5 (5.2) | 6 (6.2) | >0.999 |
| Concomitant medications | | | |
| HMG-CoA reductase inhibitors | 34 (35.1) | 28 (28.9) | 0.442 |
| Proton pump inhibitors | 31 (32.0) | 29 (29.9) | 0.878 |
| Anilides | 31 (32.0) | 26 (26.8) | 0.529 |
| Vitamin D and analogues | 25 (25.8) | 25 (25.8) | 1.000 |
| Calcium | 20 (20.6) | 19 (19.6) | >0.999 |
| Glucocorticoids | 17 (17.5) | 19 (19.6) | 0.854 |
| Platelet aggregation inhibitors excluding heparin | 20 (20.6) | 14 (14.4) | 0.345 |
| Angiotensin II antagonists, plain | 15 (15.5) | 17 (17.5) | 0.847 |
| Thyroid hormones | 17 (17.5) | 15 (15.5) | 0.847 |
| Fluoroquinolones | 11 (11.3) | 12 (12.4) | >0.999 |
| Calcium, combinations with vitamin D and/or other drugs | 14 (14.4) | 8 (8.2) | 0.257 |
| Benzodiazepine derivatives | 9 (9.3) | 12 (12.4) | 0.645 |
| Other lipid modifying agents | 10 (10.3) | 11 (11.3) | >0.999 |
| Biguanides | 10 (10.3) | 10 (10.3) | 1.000 |
| Dihydropyridine derivatives | 11 (11.3) | 9 (9.3) | 0.814 |

Data shown are number of patients (%)

*Chi-squared test or Fisher’s exact test for proportions (%)

## Figure S1. Joint effusion and swelling

P values were performed using the chi-squared test; none demonstrated statistical significance CS, chondroitin sulfate

Analyses are based on available data
